# Unveiling Species Diversity of *Plectosphaerellaceae* (*Sordariomycetes*) Fungi Involved in Rhizome and Root Rots of Ginger in Shandong Province, China

**DOI:** 10.3390/microorganisms13092180

**Published:** 2025-09-18

**Authors:** Qian Zhao, Ao Jia, Hongjuan Yang, Jinming Hu, Xuli Gao, Weiqin Zhao, Lifeng Zhou, Miao Zhang, Zhaoxia Li, Weihua Zhang

**Affiliations:** 1 Shandong Key Laboratory of Bulk Open-Field Vegetable Breeding, Ministry of Agriculture and Rural Affairs Key Laboratory of Huang Huai Protected Horticulture Engineering, Institute of Vegetables, Shandong Academy of Agricultural Sciences, Jinan 250100, China; zhao_qian90@163.com (Q.Z.); jiaao2581370132@163.com (A.J.); yanghongjuan1997@163.com (H.Y.); hujinming317@126.com (J.H.); gaoxuli518@163.com (X.G.); zwq2998311872@163.com (W.Z.); lizhaoxia880705@163.com (Z.L.); 2 College of Plant Protection, Shandong Agricultural University, Tai’an 271018, China; 3 Lanling Vegetable Industry Development Center, Linyi 277700, China; zhoulifeng1975@126.com; 4 Weifang Agricultural Technology Extension Center, Weifang 261061, China; zhangmiaojysc@163.com

**Keywords:** *Glomerellales*, *Zingiber officinale*, rhizome rot, polyphasic analysis, taxonomy, new taxa

## Abstract

Ginger holds significant economic importance in both China and worldwide agriculture. Fungi from the family *Plectosphaerellaceae* are globally recognized as aggressive plant pathogens. However, the effects of *Plectosphaerellaceae* species on ginger have been poorly understood. In this research, we identified two novel *Musidium* species (*M. shandongensis* sp. nov. and *M. zingiberis* sp. nov.), one newly recorded species (*Gibellulopsis serrae*) and one new host record (*Plectosphaerella cucumerina*) from the rotten rhizomes and roots of ginger in Shandong Province, China, utilizing morphological observations combined with multilocus phylogenetic analysis of the 28S large subunit (LSU), internal transcribed spacer (ITS) region, and translation elongation factor 1-alpha (*TEF1*-*α*) gene, along with pathogenicity analyses. Key diagnostic features include *M. shandongensis* exhibiting abundant mycelium ropes and coils, *M. zingiberis* showing dark olivaceous colonies, *G. serrae* producing brown chlamydospores, and *P. cucumerina* displaying conspicuous guttulae conidia. Comparative analyses with closely related taxa were based on detailed morphological descriptions, illustrations, and phylogenetic analyses. Artificial inoculation of healthy ginger in vitro and in vivo assays caused characteristic symptoms, such as wilt, leaf yellowing, and rhizome necrosis, identical to those observed on naturally infected plants. Our findings broaden current knowledge on the diversity of *Plectosphaerellaceae* associated with ginger, revealing them as serious threats to ginger cultivation in China.

## 1. Introduction

Ginger (*Zingiber officinale* Roscoe), which originates from Southeast Asia, is globally cultivated primarily for its underground rhizomes [1]. It contains a variety of bioactive components that exhibit potent antioxidant, antitumor, and anti-inflammatory effects [2]. Traditionally, it has been widely utilized as a vegetable, spice, and herbal medicine [3,4]. In China, the cultivation area of ginger has reached 63,806 hectares, with a production of 672,914.37 tons, representing approximately 15% of the global output (FAOSTAT 2023). Despite its high economic value, ginger remains very susceptible to multiple devastating diseases, including soft rot, bacterial wilt, rust, anthracnose, and leaf spot [5,6,7]. Notably, rhizome rot has become a serious threat to ginger cultivation, causing substantial yield losses in ginger-growing regions worldwide. According to recent studies, rhizome rot can lead to yield losses ranging from 20 to 100% [8,9,10], and the occurrence area of ginger rhizome rot has been consistently increasing [11]. A wide range of pathogens are associated with this disease, including fungi (*Fusarium oxysporum*, *Rhizoctonia solani*, *Sclerotium rolfsii*), bacteria (*Ralstonia solanacearum*, *Pectobacterium brasiliense*, *Pseudomonas* spp.), oomycetes (*Pythium* spp.), and plant-parasitic nematodes (*Meloidogyne incognita*) [12,13,14].

*Gibellulopsis*, *Musidium*, and *Plectosphaerella* belong to *Plectosphaerellaceae*, *Glomerellales* in *Sordariomycetes* (Index Fungorum 2025) [15]. The family *Plectosphaerellaceae* was established by Zare et al. [16] and currently comprises 24 genera. Species of *Plectosphaerellaceae* are globally distributed and thrive in various ecological niches. They are primarily soil-borne saprotrophs or parasites of plants, other fungi, insects, and animals [17,18,19,20,21].

*Gibellulopsis*, originally proposed by Batista & da Silva Maia [18], was revived by Zare et al. [16], with *G. piscis* as its type species. The genus *Gibellulopsis* exhibits hyaline vegetative hyphae, verticillium-like conidiophores, and pigmented intercalary or terminal chlamydospores. Currently, seven species are accepted within *Gibellulopsis*, isolated from both terrestrial and aquatic environments (Index Fungorum 2025) [15]. Most taxa occurred in soil and decaying plants, while some inhabited cloud water, human and animal tissues, water-damaged structures, and packaging materials [22]. The species *G. serrae* was named by Giraldo López & Crous [22] for a new combination based on *Cephalosporium serrae*, *Verticillium serrae*, and *Hyalopus* *serrae* as the synonyms. It is generally known as a frequent soil-borne plant pathogen, and for its salt tolerance properties [23]. To date, *G. serrae* has been reported in only Cuba, Italy, Canada, India, Germany, Israel, and Japan, but has not been recorded in China (USDA Fungal databases 2025) [24].

The monotypic genus *Musidium* was established by Giraldo López & Crous [22] to accommodate a species formerly known as *Acremonium stromaticum*, which was described based on isolates obtained from *Musa* sp. in Honduras [25]. *Musidium* is characterized by septate hyphae, poorly branched conidiophores, slimy heads conidia, and dark olivaceous stromata. The type species, *M. stromaticum*, is commonly referred to as a phytopathogen that colonizes the root, rhizosphere, and leaf of *Musa* sp. [22]. In addition, the species of *M. stromaticum* has been reported as pathogens causing brown rhizome rot in ginger [26]. To date, *M. stromaticum* has been reported in only Colombia, Costa Rica, Honduras, Panama, Philippines, Tanzania, England, and Japan (USDA Fungal databases 2025) [24].

The genus *Plectosphaerella* was proposed by Klebahn [27] on the basis of *P. cucumerina* as its type species. Previously, it was a member of the *Hypocreaceae* (*Sordariomycetes*, *Hypocreales*) [28]. It was subsequently placed in *Sordariaceae* (*Sordariomycetes*, *Sordariales*) on the basis of centrum development type [29]. Subsequently, Zare et al. [16] introduced the family *Plectosphaerellaceae* to accommodate the genus. Its asexual morph was known as *Plectosporium* [30]. Réblová et al. recommended *Plectosphaerella* rather than *Plectosporium* as the accepted generic name, given the former’s priority and extensive use in phytopathology [31]. The genus *Plectosphaerella* typically exhibits simple or sparsely branched conidiophores, phialide-like conidiogenous cells, and mostly cylindrical conidia arranged in slimy heads [16]. According to Index Fungorum (2025) [15], the genus includes 24 accepted species, although *P. himantia*, *P. kunmingensis*, and *P. melaena* are not considered for *Plectosphaerella*. Species of *Plectosphaerella* are globally distributed, occupying various ecological niches [17,32], and are commonly mentioned as fungal pathogens on diverse plants, including melon, pumpkin, ranunculus, tomato, pepper, and bamboo [33,34,35,36]. Also, some species act as endophytic fungi [37] or serve as opportunistic pathogens on arthropods [20]. The combination *P. cucumerina*, proposed by Gams [38], has been acknowledged as a ubiquitous species with a broad environmental tolerance. It is the causative agent of root rot and wilt diseases in 86 plant species from 54 genera around the world [17,22,30,35,36,37,38,39,40]. In addition, it is relevant in the clinical field [41,42] and occurs in soil, dung, and paper [30].

Although *Gibellulopsis*, *Musidium*, and *Plectosphaerella* have been well studied in recent years [17,22,43], knowledge concerning these genera within ginger remains limited. From August 2023 to December 2024, we observed ginger exhibiting symptoms of wilt, leaf yellowing, basal stem browning, rhizome rot, and root necrosis. The symptoms were similar to those caused by fungi of the family *Plectosphaerellaceae*. This study focused on the identification of new fungal taxa obtained from the rot rhizomes and roots of ginger sampled in Shandong Province, the largest ginger-producing region in China. Through a polyphasic approach combining morphological features, multilocus phylogenetic inference and pathogenicity assessment, we proposed two novel species of *Musidium*, namely *M. shandongensis* sp. nov. and *M. zingiberis* sp. nov., reported *G. serrae* as a newly recorded species in China, and identified *P. cucumerina* as a new host record infecting ginger. Detailed morphological descriptions, illustrations, and phylogenetic data of all identified species are provided. To our knowledge, this is the first identification and characterization of *Gibellulopsis*, *Musidium*, and *Plectosphaerella* species associated with ginger in China.

## 2. Materials and Methods

### 2.1. Samples Collection, Fungal Isolation, and Morphological Observations

Ginger plants exhibiting brown lesions and rot symptoms on rhizomes, roots, and stems were sampled from Anqiu City, Changle County, Laizhou City, Laiwu District, Pingdu City, and Yishui County in Shandong Province, China, from 2023 to 2024. All the sampling sites are major ginger-producing areas whose cultivation exceeds approx. 3335 hectares. The samples were carefully sealed in resealable plastic bags, properly labeled with sampling information, and subsequently transported to the laboratory.

Fungal isolation was performed using the tissue separation method. Symptomatic tissues (~25 mm^2^) were excised from the margins of the necrotic lesions following surface sterilization with 75% ethanol (30 s) and 5% NaClO (1 min), then rinsed thrice with sterile water. The sterilized tissues were dried on sterile filter paper and then transferred to potato dextrose agar (PDA; composed of 20% potato extract, 2% glucose, and 1.5% agar) Petri dishes, followed by incubation in the dark at 25 °C for 5–7 d. Pure colonies were obtained after sequential passage by hyphal tip transfer on PDA. The obtained isolates were stored on PDA slants for short-term preservation at 4 °C, and in 25% glycerol for long-term preservation at −80 °C. The specimens were deposited in the Fungal Herbarium of the Shandong Academy of Agricultural Sciences (SAAS), and corresponding living cultures were preserved in Qian Zhao’s personal culture collection (QZ).

Colony characteristics and diameters were determined from cultures grown on PDA and oatmeal agar (OA, 3% filtered oat flakes and 1.5% agar) media after incubation for 7 days at 25 °C in the dark and imaged with a digital camera (Canon EOS M100, Tokyo, Japan). Color descriptions were based on Rayner’s method [44]. The micromorphological characteristics, including shapes, colors, and sizes of hyphae, conidiophores, conidiogenous cells, and conidia, were examined and documented with lactic acid as a mountant using a biological microscope (Leica DM 1000, Wetzlar, Germany) equipped with a Leica MC 170HD digital camera. At least 30 random measurements of each structure were recorded using the Nano Measurer v. 1.2 software (Version 1.2, Shanghai, China). Newly described taxa were registered in the Fungal Names database.

### 2.2. DNA Extraction, PCR Amplification, and Sequencing

Genomic DNA was extracted from actively growing mycelia cultured on PDA using the Plant Genomic DNA Kit (Tiangen Biotech Co., Ltd., Beijing, China). The 28S large subunit (LSU) region, the internal transcribed spacer (ITS) region, and the translation elongation factor 1-alpha (*TEF1*-*α*) gene were amplified and sequenced using the corresponding primer pairs ITS4/ITS5 [45], LR0R/LR5 [46,47], and EF1-983F/EF1-2218R [48]. Polymerase chain reaction (PCR) was performed in a 25 μL mixture, comprising 12.5 μL of 2 × Taq PCR Master Mix (Biomed Biotech Co., Ltd., Beijing, China), 1 μL each of forward and reverse primers (10 μM), 1 μL of template DNA, and distilled deionized water to adjust the volume. The PCR parameters included an initial denaturation at 95 °C for 3 min, followed by 35 cycles of denaturation at 95 °C for 30 s, annealing at 50 °C for 30 s, and extension at 72 °C for 60 s, with a final elongation step at 72 °C for 10 min. The annealing temperature was optimized by performing a gradient PCR, and 50 °C was chosen as it consistently yielded specific, strong amplification products. Amplified products were visualized on 1% agarose electrophoresis gels and sequenced by Tsingke Biotech Co., Ltd. (Beijing, China).

### 2.3. Phylogenetic Analyses

Consensus sequences newly generated in this study were assembled using DNAMAN v. 7.0 (Lynnon Biosoft, San Ramon, CA, USA) and deposited in GenBank. Additional reference sequences were downloaded from GenBank (https://www.ncbi.nlm.nih.gov/genbank, accessed on 10 June 2025). Details for all sequences are provided in Table 1. Individual gene alignments were conducted using MAFFT v. 7 (http://mafft.cbrc.jp/alignment/server/index.html, accessed on 10 June 2025) [49] with default settings and then manually adjusted in MEGA v. 7 [50]. Phylogenetic analysis was performed based on concatenated LSU, ITS, and TEF1-α loci using Bayesian inference (BI) and maximum likelihood (ML) methods. BI analysis was conducted using MrBayes v. 3.2.1 [51]. The best-fit models of nucleotide substitution for each partition were determined by MrModeltest v. 2.3 [52] under the Akaike Information Criterion (AIC). All characters were assigned equal weights, and alignment gaps were considered as missing data. Four simultaneous Markov Chain Monte Carlo (MCMC) chains were run for 1 million generations, sampling every 1000 generations, and stopping when the mean standard deviation of split frequencies was less than 0.01. The first 25% of sampled trees were removed as burn-in, and the posterior probability (PP) above 0.95 was considered significant [53]. ML analysis was conducted in IQ-TREE v1.6.8 [54], using optimal nucleotide substitution models determined by PartitionFinder v2.0 [55]. The branch support estimation utilized a bootstrapping (BS) method with 5000 replicates [56]. Bootstrap values over 70% were considered significant [49]. The resulting trees were visualized using FigTree v. 1.4.3 [57]. Both the BI and ML trees were rooted with *Monilochaetes infuscans* CBS 379.77.

**Table 1:** List of species, collections, and sequences used in the phylogenetic analyses in Figure 1.

Species	Voucher/Culture	GenBank Accession Number		
		LSU	ITS	*TEF1*-*α*
*Acremonium acutatum*	CBS 140.62	OQ055348	OQ429437	OQ470734
	CBS 682.71^T^	NG_056976	NR_163811	OQ470735
	CBS 829.73	OQ055350	OQ429439	OQ470736
*A. aerium*	CBS 189.70^T^	OQ055352	NR_189420	OQ470738
	CBS 379.70C	OQ055351	OQ429440	OQ470737
*A. alternatum*	CBS 407.66^T^	NG_056977	NR_144913	OQ470739
*A. brunneisporum*	CBS 413.76^T^	NG_243194	NR_190249	OQ470741
*A. chlamydosporium*	CBS 414.76^T^	OQ055361	NR_189421	OQ470748
*A. multiramosum*	CBS 147436^T^	NG_242036	NR_189426	OQ470770
*A. sordidulum*	CBS 385.73^T^	NG_056992	NR_159618	OQ470782
*Gibellulopsis aquatica*	CBS 117131^T^	LR025850	LR026720	LR026414
*G. catenata*	CBS 113951^T^	LR025851	LR026721	LR026415
*G. fusca*	CBS 308.38	LR025852	LR026722	LR026416
	CBS 560.65^T^	LR025854	LR026724	LR026418
	CBS 120818	LR025856	LR026726	LR026420
*G. nigrescens*	CBS 470.64	LR025860	LR026730	LR026422
	CBS 100829	LR025862	LR026732	LR026423
	CBS 120949^NT^	LR025868	LR026738	LR026429
***G. serrae***	CBS 290.30^T^	LR025872	LR026742	LR026433
	CBS 383.66	LR025874	LR026744	LR026435
	CBS 892.70^T^	LR025885	LR026755	LR026445
	CBS 100826	LR025886	LR026756	LR026446
	**SAAS 311704**	**PV702888**	**PV702874**	**PV701791**
	**SAAS 410805**	**PV702887**	**PV702873**	**PV701790**
*Monilochaetes infuscans*	CBS 379.77	GU180645	LR026764	LR026460
***Musidium shandongensis***	**SAAS 381414^T^**	**PV702883**	**PV702869**	**PV701786**
	**SAAS 403027**	**PV702884**	**PV702870**	**PV701787**
*M. stromaticum*	CBS 132.74	LR025919	LR026785	LR026479
	CBS 133.74	LR025920	LR026786	LR026480
	CBS 135.74A	LR025922	LR026787	LR026482
	CBS 135.74C	LR025923	LR026788	LR026483
	CBS 135.74F	LR025925	LR026790	LR026484
	CBS 863.73^T^	HQ232143	DQ825969	LN810533
***M. zingiberis***	**SAAS 381402^T^**	**PV702881**	**PV702867**	**PV701784**
	**SAAS 442806**	**PV702882**	**PV702868**	**PV701785**
*Plectosphaerella alismatis*	CBS 113362^T^	LR025932	LR026794	LR026489
*P. citrullae*	CBS 131740	LR025933	LR026795	LR026490
	CBS 131741^T^	LR025934	LR026796	LR026491
***P. cucumerina***	CBS 137.33^NT^	LR025935	LR026797	LR026492
	CBS 137.37^T^	LR025936	LR026798	LR026493
	CBS 101014	LR025945	LR026807	LR026502
	CBS 131739^NT^	LR025947	LR026809	LR026504
	**SAAS 311708**	**PV702886**	**PV702872**	**PV701789**
	**SAAS 481921**	**PV702885**	**PV702871**	**PV701788**
*P. delsorboi*	CBS 116708^T^	LR025948	LR026810	LR026505
*P. melonis*	CBS 489.96^T^	LR025950	LR026812	LR026507
	CBS 525.93	LR025951	LR026813	LR026508
*P. populi*	CBS 139623^T^	KR476783	KR476750	LR026527
	CBS 139624	MH878144	KR476751	LR026528
*P. ramiseptata*	CBS 131743	LR025969	LR026831	LR026529
	CBS 131861^T^	LR025970	LR026832	LR026530
*Verticillium alboatrum*	CBS 130340^ET^	LR025984	LR026847	LR026543
*V. alfalfae*	CBS 130603^T^	LR025988	LR026851	LR026547
*V. dahliae*	CBS 127.79B	LR025989	LR026852	LR026548
	CBS 179.66	LR025992	LR026854	LR026549
	CBS 384.49	LR026000	LR026861	LR026554
*V. nonalfalfae*	CBS 113707	LR026071	LR026932	LR026587
	CBS 130339^T^	LR026074	LR026935	LR026590
*V. nubilum*	CBS 457.51^T^	LR026076	LR026937	LR026591
*V. tricorpus*	CBS 126.79	LR026078	LR026939	LR026592
	CBS 255.57	LR026081	LR026942	LR026595
*V. zaregamsianum*	CBS 130342^T^	LR026098	LR026959	LR026610

The ex-type, ex-epitype and ex-neotype are indicated using “T”, “ET” and “NT” after strain numbers, and newly generated sequences are indicated in bold.

**Figure 1:**
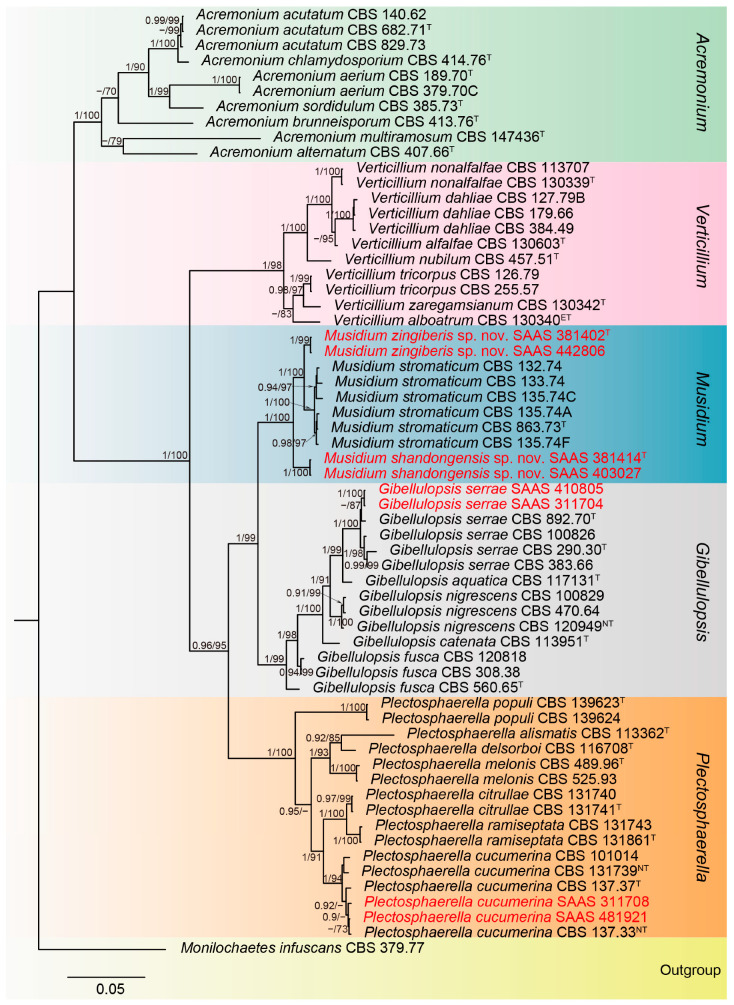
Phylogenetic tree of *Plectosphaerellaceae* generated by Bayesian analysis based on concatenated sequences of the LSU, ITS and *TEF1*-*α* loci. The tree was rooted to *Monilochaetes infuscans* (CBS 379.77). Bayesian posterior probability (PP ≥ 0.90) and RAxML bootstrap support values (ML ≥ 70%) were shown at the nodes (PP/ML BS). Strains marked with “T”, “ET” and “NT” are ex-type, ex-epitype, and ex-neotype, respectively. Newly generated sequences are indicated in red.

### 2.4. Pathogenicity Test

Pathogenicity tests were performed on all *Plectosphaerellaceae* species isolated from ginger to fulfill Koch’s postulates, as none of them have been previously documented as causal agents of ginger rhizome rot.

In vitro *assay*: Rhizomes from ginger cv. Laiwu Dajiang were surface-sterilized in 20% ethanol for 30 s, then placed in Petri dishes containing a single layer of moistened sterile filter paper. For each fungal isolate, 5 mm-diameter mycelial disks were inoculated onto wounds created on the rhizomes using a sterile cork borer. The mycelial disks were obtained from 7-day-old colonies of *G. serrae* (SAAS 311704), *M. shandongensis* (SAAS 381414), *M. zingiberis* (SAAS 381402), and *P. cucumerina* (SAAS 481921). Control rhizomes were similarly treated but inoculated with sterile PDA disks. Each fungal strain was tested on nine replicate rhizomes. After incubation at 25 ± 2 °C for 7 days, disease severity was assessed using a 0 to 5 scale as follows: 0 = no symptoms; 1 = 1–20%; 2 = 21–40%; 3 = 41–60%; 4 = 61–80%; and 5 = 81–100% of the rhizome surface exhibiting necrosis. The disease severity index (DSI) was calculated as: DSI = [Σ(number of rhizomes in each disease class × class score)/(total number of rhizomes × maximum disease score)] × 100.

In vivo *assay*: Two-month-old seedlings of ginger cv. Laiwu Dajiang were transplanted into 628 mL pots containing sterilized commercial potting substrate. Seven days after transplanting, each plant was inoculated via soil drenching with 5 mL of a conidial suspension (10^6^ conidia mL^−1^), prepared from 10-day-old cultures of each tested fungal species. Plants treated with sterile water served as negative controls. Each fungal isolate was tested on nine seedlings. The seedlings were maintained in a greenhouse (25 ± 2 °C, 70% relative humidity, natural light) for 14 days. Subsequently, rhizomes were removed from pots, carefully washed, and scored for browning symptoms using the 0 to 5 scale described above. To satisfy Koch’s postulates, the fungi were re-isolated from symptomatic rhizomes.

## 3. Results

### 3.1. Phylogenetic Analysis

Among the isolates obtained, strains SAAS 311704, SAAS 381402, SAAS 381414, and SAAS 481921 exhibited the characteristic morphological features of *Plectosphaerellaceae*. BLAST (v. 2.14.0) analyses of LSU, ITS, and *TEF1*-*α* sequences (Table 2) further confirmed the taxonomic affiliation of these strains to this family.

To resolve phylogenetic relationships, a concatenated sequence alignment of LSU, ITS, and *TEF1*-*α* loci was generated, comprising sixty-two isolates representing six genera: *Acremonium* (ten sequences), *Verticillium* (eleven sequences), *Musidium* (ten sequences), *Gibellulopsis* (fourteen sequences), *Plectosphaerella* (sixteen sequences), and *Monilochaetes infuscans* CBS 379.77 (used as the outgroup). The final dataset contained 2082 characters, including gaps (LSU: 756 bp; ITS: 539 bp; TEF-1α: 787 bp). Of these, 646 characters were variable sites (LSU: 179 bp; ITS: 236 bp; TEF-1α: 231 bp), and 530 characters were parsimony informative (LSU: 149 bp; ITS: 190 bp; TEF-1α: 191 bp). BI analysis was conducted using the best-fit substitution model GTR+I+G across all loci. For ML analysis, the GTR+I+G model was applied to LSU and *TEF1*-*α*, while GTR+G was selected for ITS. The topology of multilocus phylogenetic trees obtained from BI and ML inference analyses was congruent; thus, the Bayesian tree is shown (Figure 1). Phylogenetic analyses indicated that the strains of *M. zingiberis* (SAAS 381402 and SAAS 442806) formed an independent, well-supported branch (1.0 PP, 99% BS) sister to a lineage containing *M. stromaticum*. In addition, *M. shandongensis* strains (SAAS 381414 and SAAS 403027) clustered into a separate, strongly supported clade (1.0 PP, 100% BS) closely related to *M. zingiberis* and *M. stromaticum*. Moreover, the new *G. serrae* strains (SAAS 311704 and SAAS 410805) grouped with ex-type strains CBS 290.30 and CBS 892.70 (1.0 PP, 100% BS). The newly obtained strains of *P. cucumerina* (SAAS 311708 and SAAS 481921) grouped with ex-type strain CBS 137.37 and ex-neotype strains CBS 137.33 and CBS 131739 (1.0 PP, 94% BS). Overall, our isolates represent four phylogenetically distinct lineages, corresponding to two previously known species and two novel species.

### 3.2. Taxonomy

#### 3.2.1. Gibellulopsis serrae (Maffei) Giraldo López & Crous, Stud Mycol 92: 250 (2018) Figure 2

Fungal Names number: FN 828040.

Materials examined: China, Shandong Province, Qingdao City, Pingdu City, Mingcun Town, Xinan Street, 36°45′36″ N, 119°38′50″ E, 53 m asl, on rhizomes and roots of *Zingiber officinale* (*Zingiberaceae*), 7 November 2023, Q. Zhao, H.J. Yang, J.M. Hu, XAJ4; ibid., XAJ16. Yantai City, Laizhou City, Pinglidian Town, 37°17′22″ N, 120°1′31″ E, 27 m asl, on rhizomes of *Zingiber officinale* (*Zingiberaceae*), 8 October 2024, Q. Zhao, H.J. Yang, A. Jia, PLD5. Yantai City, Laizhou City, Yidao Town, 37°13′58″ N, 120°10′32″ E, 78 m asl, on rhizomes of *Zingiber officinale* (*Zingiberaceae*), 5 December 2024, Q. Zhao, H.J. Yang, A. Jia, YDZ13.

*Mycelium* consists of branched, septate, hyaline hyphae with smooth and thin walls, 1.6–2.7 μm in width. *Conidiophores* arise from either aerial or submerged hyphae, solitary or aggregated, erect or slanted, straight or irregularly curved, unbranched or irregularly branched, bearing 1–2 levels of 1–4 phialides at each node, hyaline, smooth-walled, generally possessing thicker walls than the vegetative hyphae, reaching 45–74 μm in length and 1.8–3.2 μm in basal width. *Conidiogenous cells* are enteroblastic, monophialidic, terminal or lateral, long-cylindrical to aculeate, hyaline, smooth-walled, with a minute cylindrical collarette and inconspicuous periclinal thickening at the conidiogenous locus, 23–51 μm long and 1.4–2.8 μm wide at the base. *Conidia* are unicellular, straight, hyaline, thin-walled, displaying morphologies from elongate ellipsoidal to cylindrical with rounded ends, sometimes containing two inconspicuous guttulae, 3.3–5.5 × 1.4–2.6 μm. *Chlamydospores* are intercalary or terminal, appearing singly or in short chains, subglobose to clavate, thick-walled, gray-brown to dark brown, 5.8–7.9 × 3.1–5.4 μm. *Sexual morph* was not observed.

Culture characteristics: After 7 days of incubation at 25 °C, colonies on PDA reached 27–29 mm in diameter, exhibiting a flat morphology with moderate, slightly cottony aerial mycelium and white surface appearance. The colony reverse showed an apricot center, gradually fading to creamy white toward the margins. On OA, colonies grew to 24–27 mm in diameter, appearing smooth, felty, membranous with scarce aerial mycelium, hyaline to light gray at the center and pale white at the margin; the reverses were concolorous.

Notes: *G. serrae* forms a strongly supported clade (1.0 PP, 100% BS) closely related to *G. aquatica*, *G. nigrescens*, and *G. catenata*. Morphologically, *G. serrae*, *G. aquatica*, and *G. nigrescens* produce exclusively 1-celled conidia, while *G. catenata* may produce both 1- and 2-celled conidia. Conidial dimensions for *G. serrae* (3.3–5.5 × 1.4–2.6 μm) are shorter than those of *G. catenata* (4.1–12.9 × 1.5–2.8 μm), and also slightly shorter than those of *G. aquatica* (3.9–6.1 × 1.6–2.5 μm) and *G. nigrescens* (4.1–5.6 × 1.6–2.3 μm). Conidiophores further distinguish *G. serrae*, which bears 1–2 verticillate levels with 1–4 phialides per node, from *G. aquatica* (1–6 levels, 1–2 phialides) and *G. nigrescens* (1–4 levels, 1–3 phialides). Additionally, conidiophores of *G. serrae* (up to 74 μm) are shorter than those of *G. aquatica* (104 μm) and *G. nigrescens* (100 μm). Moreover, *G. serrae* can be distinguished from *G. nigrescens* by the conidia color (hyaline vs. pale brown with age) and the chlamydospores shape (short chains vs. single). *G. serrae* has thus far been recorded only from Canada, Cuba, Germany, India, Israel, Italy, and Japan. Consequently, *G. serrae* is reported for the first time in China.

**Figure 2:**
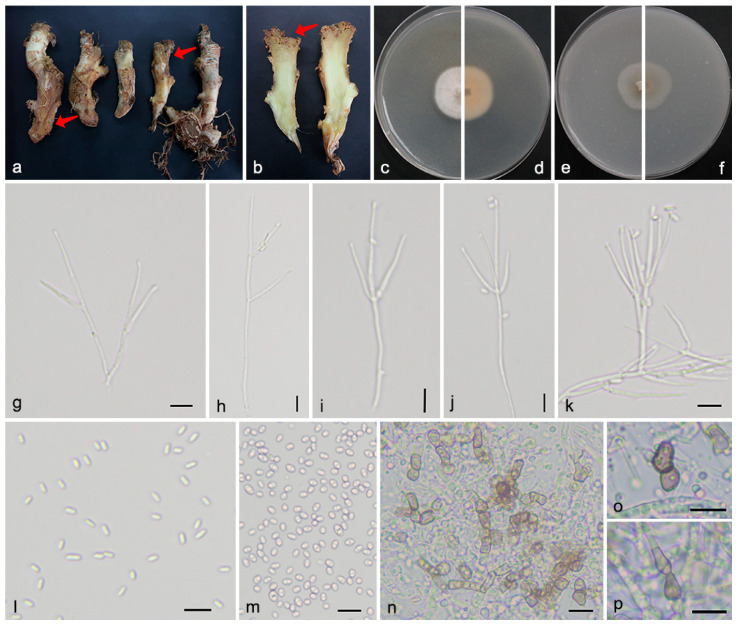
*Gibellulopsis serrae* (SAAS 311704). (**a**,**b**) Diseased rhizomes of *Zingiber officinale*. The arrows indicate the disease lesions; (**c**,**d**) surface and reverse sides of colony after 7 days on PDA (**e**,**f**) and on OA; (**g**–**k**) conidiophores; (**l**,**m**) conidia; and (**n**–**p**) chlamydospores. Scale bars: (**g**–**p**) 10 µm.

#### 3.2.2. *Musidium shandongensis* Q. Zhao & W.H. Zhang, sp. nov. Figure 3

Fungal Names number: FN 572230.

Etymology: *shandongensis* refers to the collection site, Shandong Province, China.

Materials examined: China, Shandong Province, Jinan City, Laiwu District, Gaozhuang Town, Dongwennan Village, 36°10′44″ N, 117°37′1″ E, 185 m asl, on stems and rhizomes of *Zingiber officinale* (*Zingiberaceae*), 14 August 2023, Q. Zhao, H.J. Yang, J.M. Hu, DWN4 (holotype SAAS 381414, ex-type living culture QZ 23829); ibid., DWN23. Weifang City, Xiashan District, Wangjiazhuang Town, Dashuangguotou Village, 36°30′15″ N, 119°22′29″ E, 31 m asl, on rhizomes of *Zingiber officinale* (*Zingiberaceae*), 30 October 2024, Q. Zhao, H.J. Yang, A. Jia, DSG3, DSG7; ibid., DSG14, DSG16.

**Figure 3:**
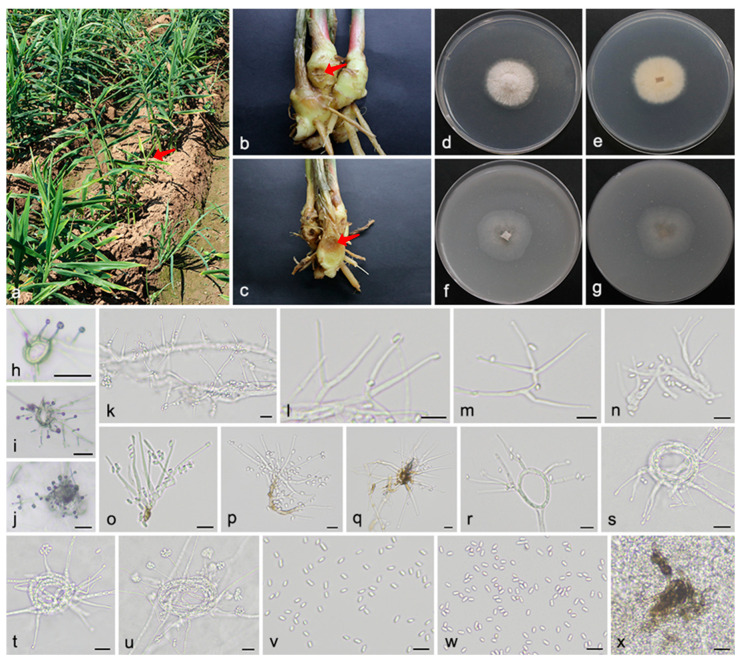
*Musidium shandongensis* (SAAS 381414). (**a**–**c**) Diseased plants, rhizomes and roots of *Zingiber officinale*. The arrows indicate the disease lesions; (**d**,**e**) surface and reverse sides of colony after 7 days on PDA (**f**,**g**) and on OA; (**h**–**j**) conidiophores with conidial heads on mycelial coils; (**k**–**q**) conidiophores; (**r**,**s**) mycelial coils; (**t**,**u**) conidiophores with conidia arranged in slimy heads; (**v**,**w**) conidia; and (**x**) stromatic hyphae. Scale bars: (**h**–**j**) 100 µm; and (**k**–**x**) 10 µm.

*Mycelium* consists of hyaline, branched, septate hyphae with smooth and thin walls, often producing abundant mycelial ropes and coils, 1.5–2.3 μm in width. *Conidiophores* emerge either individually or in clusters from aerial, substratal, or rope and coiled hyphae. They are straight to flexuous, unbranched or basitonously branched, hyaline, smooth- walled, usually possessing thicker walls than the vegetative hyphae, reaching up to 59.9 μm in length and 3.0 μm in width at the base. *Conidiogenous cells* are enteroblastic and monophialidic, positioned either terminally or laterally, and exhibit a subulate or acicular shape. They are hyaline, smooth-walled, and feature a short cylindrical collarette along with a prominent periclinal thickening, 16.3–52.3 × 2.0–3.0 μm. *Conidia* are unicellular, straight, cylindrical to ellipsoidal with rounded ends, hyaline, smooth- and thin-walled, typically produced in slimy heads, 4.0–5.3 × 2.1–3.0 μm. *Stromatic* hyphae are dark olivaceous, thick-walled, smooth, and formed at the base of plate cultures or along the margins of agar slants. *Sexual morph* was not observed.

Culture characteristics: On PDA, colonies reached 31–34 mm in diameter after 7 days at 25 °C, presenting as flat, wooly, dirty white, with fimbriate margins, and creamy-white reverses. On OA, colonies grew to 31–36 mm in diameter, with flat, membranous surfaces, scarce aerial mycelium, dirty white centers, pale gray peripheries, filiform margins, and concolorous reverses.

Notes: This type of strain of *M. shandongensis* formed a distinct lineage sister to the clade harboring *M. stromaticum* and *M. zingiberis* (1.0 PP, 100% BS). Morphologically, *M. shandongensis* can be distinguished from *M. stromaticum* by the formation of abundant mycelial ropes and coils as well as shorter and wider conidia (4.0–5.3 × 2.1–3.0 vs. 4.2–6.2 × 1.4–2.3 μm). Compared to *M. zingiberis*, *M. shandongensis* features more slender conidiophores (59.9 × 3.0 vs. 52.6 × 3.5 μm), narrower conidiogenous cells (16.3–52.3 × 2.0–3.0 vs. 16.5–43.7 × 2.1–3.8 μm), and smaller conidia (4.0–5.3 × 2.1–3.0 vs. 4.1–6.7 × 2.6–3.6 μm). Distinct colony color also differentiates between the two species: *M. shandongensis* forms dirty intoite and dirty white to pale gray colonies on PDA and OA, whereas *M. zingiberis* produces dark gray and dark olivaceous to pale gray colonies. The combined phylogenetic and morphological differences support the classification of *M. shandongensis* as a novel species within *Musidium*.

#### 3.2.3. *Musidium zingiberis* Q. Zhao & W.H. Zhang, sp. nov. Figure 4

Fungal Names number: FN 572825.

Etymology: *zingiberis* is named after the host genus from which it was collected, *Zingiber*.

Materials examined: China, Shandong Province, Jinan City, Laiwu District, Gaozhuang Town, Dongwennan Village, 36°10′44″ N, 117°37′1″ E, 185 m asl, on stems and rhizomes of *Zingiber officinale* (*Zingiberaceae*), 14 August 2023, Q. Zhao, H.J. Yang, J.M. Hu, DWN2 (holotype SAAS 381402, ex-type living culture QZ 23825); ibid., DWN19. Weifang City, Changle County, Yingqiu Town, Lijia Village, 36°35′11″ N, 119°3′15″ E, 56 m asl, on rhizomes of *Zingiber officinale* (*Zingiberaceae*), 28 April 2024, Q. Zhao, H.J. Yang, J.M. Hu, LJC6; ibid., LJC14. Weifang City, Xiashan District, Wangjiazhuang Town, Dashuangguotou Village, 36°30′15″ N, 119°22′29″ E, 31 m asl, on rhizomes of *Zingiber officinale* (*Zingiberaceae*), 30 October 2024, Q. Zhao, H.J. Yang, A. Jia, DSG5; ibid., DSG17.

*Mycelium* consists of branched, septate, hyaline hyphae with smooth and thin walls, frequently forming abundant coils and gracile ropes, 1.6–2.7 μm in width. *Conidiophores* arise singly or in aggregates from submerged or superficial hyphae, often radiating out from the ropes and coils. They are erect, straight to flexuous at the base, unbranched or mostly with 1–2 lateral branches, hyaline, with smooth cell walls thicker than those of vegetative hyphae, reaching up to 52.6 μm in length and 3.5 μm in width. *Conidiogenous cells* are enteroblastic, monophialidic, terminal or lateral, subulate or tapering at the top, hyaline, smooth-walled, featuring a distinct periclinal thickening and a cylindrical collarette, 16.5–43.7 × 2.1–3.8 μm. *Conidia* are aseptate, straight, cylindrical to ellipsoidal with rounded ends, hyaline, thin- and smooth-walled, and typically aggregated in slimy heads, 4.1–6.7 × 2.6–3.6 μm. *Stromatic* hyphae are dark olivaceous, smooth, branched or unbranched, thick-walled or incrusted, and produced at the base of plate cultures or along the margins of agar slants. *Sexual morph* was not observed.

Culture characteristics: After 7 days of incubation at 25 °C on PDA, colonies reached 23–27 mm in diameter, appearing flat, felty, dark gray with filiform margins and dark olivaceous reverses. On OA, colonies grew to 25–27 mm in diameter, with flat, membranous surfaces, sparse aerial mycelium, centrally dark olivaceous with pale gray peripheries, margins fimbriate, and reverses concolorous.

**Figure 4:**
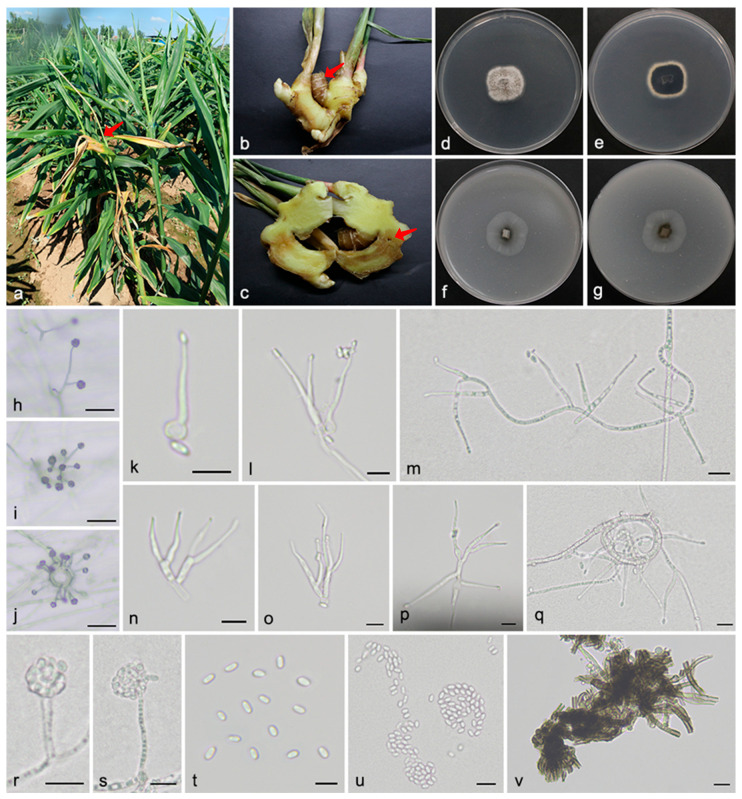
*Musidium zingiberis* (SAAS 381402). (**a**–**c**) Diseased plants and rhizomes of *Zingiber officinale*. The arrows indicate the disease lesions; (**d**,**e**) surface and reverse sides of colony after 7 days on PDA (**f**,**g**) and on OA; (**h**–**j**) conidiophores with conidial heads on mycelial ropes and coils; (**k**–**p**) conidiophores; (**q**) mycelial coils; (**r**,**s**) conidiophores with conidia arranged in slimy heads; (**t**,**u**) conidia; and (**v**) stromatic hyphae. Scale bars: (**h**–**j**) 100 µm; and (**k**–**v**) 10 µm.

Notes: The type culture of *M. zingiberis* formed a single well-supported clade (1.0 PP, 99% BS) closely related to *M. stromaticum*. Morphologically, *M. zingiberis* differs from *M. stromaticum* in its abundant mycelial coils and gracile ropes, as well as wider conidiophores, conidiogenous cells, and conidia (52.6 × 3.5 vs. 59 × 2.5 μm; 16.5–43.7 × 2.1–3.8 vs. 23–55 × 2–2.5 μm; 4.1–6.7 × 2.6–3.6 vs. 4.2–6.2 × 1.4–2.3 μm). Furthermore, colony color on PDA and OA serves as a key distinguishing feature. *M. zingiberis* produces colonies ranging from dark gray and dark olivaceous to pale gray, whereas *M. stromaticum* forms uniformly dirty white colonies. Therefore, the combination of unique morphological traits and multilocus phylogenetic evidence supports the recognition of *M. zingiberis* as a novel species in *Musidium*.

#### 3.2.4. *Plectosphaerella cucumerina* (Lindf.) W. Gams, in Domsch & Gams, Fungi in Agricultural Soils: 160 (1972) Figure 5

Fungal Names number: FN 320609.

Materials examined: China, Shandong Province, Qingdao City, Pingdu City, Mingcun Town, Xinan Street, 36°45′36″ N, 119°38′50″ E, 53 m asl, on rhizomes and roots of *Zingiber officinale* (*Zingiberaceae*), 7 November 2023, Q. Zhao, H.J. Yang, J.M. Hu, XAJ8. Linyi City, Yishui County, Yuandongtou Town, Nanqiangyu Village, 35°43′50″ N, 118°22′6″ E, 247 m asl, on rhizomes of *Zingiber officinale* (*Zingiberaceae*), 19 August 2024, Q. Zhao, H.J. Yang, A. Jia, NQY5, NQY8; ibid., NQY18, NQY21. Weifang City, Anqiu City, Xingan Town, 36°18′39″ N, 119°9′42″ E, 85 m asl, on rhizomes of *Zingiber officinale* (*Zingiberaceae*), 29 August 2024, Q. Zhao, H.J. Yang, A. Jia, XAT3.

*Mycelium* consists of branched, septate, hyaline hyphae with smooth and thin walls, occasionally forming coils, 1.8–3.0 μm in width. *Conidiophores* are mostly solitary, erect or sinuous, arising from superficial and submerged hyphae, or from hyphal coils. They are typically unbranched, rarely branched, hyaline, and thin-walled, reaching up to 55.4 μm in length and 3.8 μm in width at the base. *Conidiogenous cells* are enteroblastic, monophialidic, terminal or lateral, discrete, subcylindrical to ampulliform, occasionally sinuous, straight at the apex, gradually tapering to the apex. They are hyaline, smooth-walled, with distinct periclinal thickening and a cylindrical collarette, 11.5–37 μm in length and 1.9–3.6 μm in width at the base. *Conidia* are acrogenous, unicellular, fusiform or ellipsoidal, hyaline, straight, thin- and smooth-walled, often containing 1–5 conspicuous guttulae, 5.3–8.6 × 2.2–3.5 μm. Neither *chlamydospores* nor *sexual morphs* were observed.

Culture characteristics: After 7 days of incubation at 25 °C on PDA, colonies reached 44–46 mm in diameter, appearing flat and repressed, with slimy surfaces and sparse aerial hyphae. The colony center was buff, fading to milky white at the periphery, with a regular margin and concolorous reverse. On OA, colonies grew to 42–46 mm in diameter, presenting as flat with sparse aerial hyphae, creamy white, with a regular margin and concolorous reverse.

Notes: On the basis of multilocus phylogenetic analyses, the isolates of *P. cucumerina* clustered in a single branch (1.0 PP, 94% BS), including the ex-type *P. cucumerina* CBS 137.37 and the neotypes of *P. cucumerina* CBS 131739 and CBS 137.33, which were placed basal to the clade (1.0 PP, 91% BS) containing *P. citrullae* and *P. ramiseptata*. The conidia of *P. cucumerina* are often fusiform or ellipsoidal with up to 5 guttulae, and those of *P. citrullae* are ellipsoids with up to 2 guttulae. *P. ramiseptata* is morphologically differentiated from *P. cucumerina* by its branched and septate conidiogenous cells as well as the production of 1-septate conidia. *P. cucumerina* was first reported in China from *Lycopersicon esculentum* [58]. Subsequently, its host range extended to *Helianthus annuus*, *Lagenaria siceraria*, *Solanum tuberosum*, *Brassica oleracea*, *Cucumis sativus*, *Foeniculum vulgare*, *Raphanus sativus*, *Phaseolus vulgaris*, and *Medicago sativa* [40,59,60,61,62,63,64]. The present study represents the first identification of *P. cucumerina* isolated from ginger.

**Figure 5:**
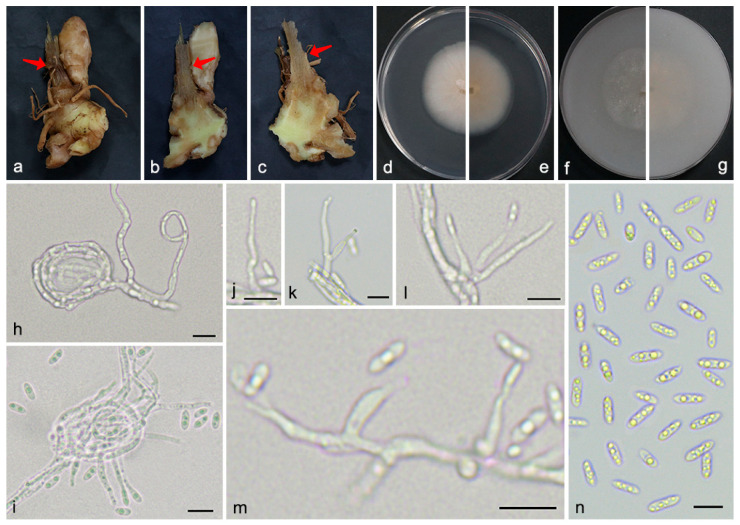
*Plectosphaerella cucumerina* (SAAS 481921). (**a**–**c**) Diseased rhizomes and roots of *Zingiber officinale*. The arrows indicate the disease lesions; (**d**,**e**) surface and reverse sides of colony after 7 days on PDA (**f**,**g**) and on OA; (**h**) mycelial coils; (**i**) conidiophores radiating out from mycelial coils; (**j**–**m**) conidiophores; and (**n**) conidia. Scale bars: (**h**–**n**) 10 µm.

### 3.3. Pathogenicity Tests

**Figure 6:**
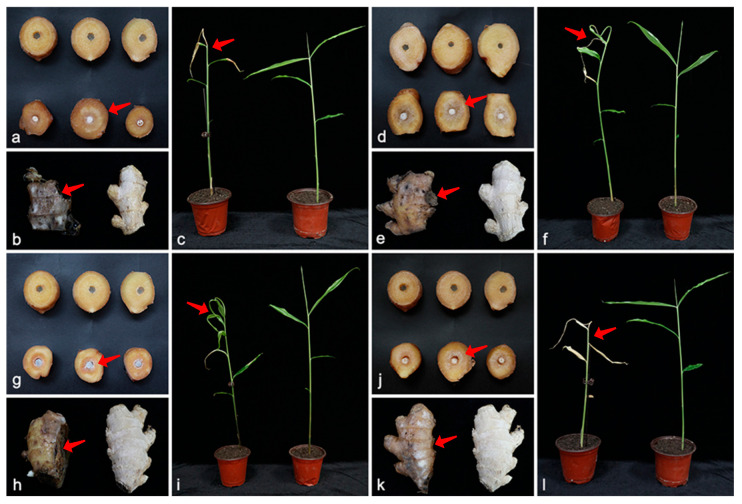
Pathogenicity assays on ginger rhizomes and seedlings for the *Plectosphaerellaceae* species. Disease symptoms on the rhizomes and seedlings caused by (**a**–**c**) *Gibellulopsis serrae* (SAAS 311704), (**d**–**f**) *Musidium shandongensis* (SAAS 381414), (**g**–**i**) *Musidium zingiberis* (SAAS 381402), and (**j**–**l**) *Plectosphaerella cucumerina* (SAAS 481921). The arrows indicate the disease lesions. (**a**,**d**,**g**,**j**) upper: the rhizomes were inoculated with sterile PDA disks as a negative control; lower: the rhizomes were inoculated with mycelial disks of the indicated fungal isolates. (**b**,**e**,**h**,**k**) left: the rhizomes were inoculated with conidial suspension of the indicated fungal isolates; right: the rhizomes were inoculated with sterile water as a negative control. (**c**,**f**,**i**,**l**) left: the seedlings were inoculated with conidial suspension of the indicated fungal isolates; right: the seedlings were inoculated with sterile water as a negative control.

In vitro *assay*: at 7 days post inoculation (dpi), uninoculated rhizomes remained healthy. Rhizomes inoculated with the four *Plectosphaerellaceae* spp. developed brown necrotic lesions (Figure 6), with DSI values ranging from 51.1 for *M. zingiberis*, to 68.9 for *M. shandongensis*, 75.6 for *P. cucumerina*, and 97.8 for *G. serrae*. Furthermore, white aerial mycelia were visible on the surface of the browning rhizomes. These symptoms closely resembled those initially observed on the source rhizomes. In vivo *assay*: at 14 dpi, control seedlings remained healthy. Seedlings inoculated with the four *Plectosphaerellaceae* spp. showed symptoms including wilt, leaf yellowing, and rhizome browning and necrosis (Figure 6), with DSI values ranging from 60.0 for *M. zingiberis*, to 71.1 for *M. shandongensis*, 77.8 for *P. cucumerina*, and 100.0 for *G. serrae*. Specifically, *G. serrae* infection resulted in bright orange leaf discoloration, defoliation, rhizome rot, and rapid plant death. *M. shandongensis* and *M. zingiberis* induced symptoms including leaf twisting-wilting and rhizome brown rot. Ginger seedlings inoculated with *P. cucumerina* exhibited light leaf yellowing, brown rhizome decay, and yellow-brown necrosis of the basal stem and root. These symptoms were identical to those observed in the field. All of these *Plectosphaerellaceae* spp. were re-isolated from symptomatic ginger rhizomes and seedlings, thereby establishing the causal link between these isolated fungi and the observed disease symptoms.

## 4. Discussion

This study investigated the diversity of *Plectosphaerellaceae* species associated with ginger rhizome and root rots in Shandong Province, China. Based on multi-locus phylogenetic analyses of concatenated LSU, ITS and *TEF1*-*α* sequences combined with morphological characteristics and pathogenicity tests, we identified four species: two new species (*Musidium shandongensis* sp. nov. and *M. zingiberis* sp. nov.), one newly recorded species (*Gibellulopsis serrae*) in China, and one new host record (*Plectosphaerella cucumerina*) on ginger.

The four species introduced in this paper all formed distinct clades in the LSU-ITS-*TEF1*-*α* phylogeny, and all were supported by morphological differences that distinguish them. BLAST analyses of the *TEF1*-*α* gene yielded identifications that differed from those of the LSU and ITS regions (Table 2), as *TEF1*-*α* is known to provide higher resolution for species in *Plectosphaerellaceae* than do the LSU or ITS loci [17,35]. The relatively high variability of *TEF1*-*α* within the *Plectosphaerellaceae* clade has already been indicated by fine interspecific distinctions [16]. Multi-locus phylogenetic analyses are necessary in delimitation of the various *Plectosphaerellaceae* species, since no single locus can resolve all known species [22,26,32,37].

*Gibellulopsis serrae* was proposed as a novel combination to accommodate *Cephalosporium serrae*, *Hyalopus serrae* and *Verticillium serrae*, which formed a well-supported clade distinct from *G. nigrescens* based on multilocus phylogenetic analysis of LSU, ITS, *TEF1*-*α* and *RPB2* regions [22]. This species has been previously isolated from diverse hosts, including *Abelmoschus esculentus* in Cuba, *Amaranthus tricolor* in Italy, *Beta vulgaris* in Canada, *Musa* sp. in India, and *Solanum tuberosum* in Germany, Israel, and Japan [22]. Furthermore, *G. serrae* was isolated from soil (Argentina, Israel and New Zealand), granulomas in goldfish (Brazil), human blood (Greece), the human eye (Italy), and wood pulp (Sweden). Interestingly, it has been recognized as a fungicolous species parasitizing *Cercospora beticola* in Moldavia, *Erysiphe* sp. in Russia, and *Oidium* sp. in Odessa [22]. No previous reports of this species associated with any plant host have been published in China. In our study, strains SAAS 311704 and SAAS 410805 clustered with ex-type strains CBS 290.30 and CBS 892.70 (Figure 1), forming a highly supported clade (1.0 PP, 100% BS). We present the first report of *G. serrae* from China, a new geographical record.

The genus *Musidium* was introduced for the species originally known as *Acremonium stromaticum* [22]. Only one species, *M. stromaticum*, has thus far been accepted in the genus. This species has been documented in *Musa* sp. mainly in Central America and Europe [22], as well as in *Zingiber officinale* in India and Japan [25,26]. In the present study, two new *Musidium* species (*M. shandongensis* and *M. zingiberis*) were introduced based on both multilocus phylogenetic analyses (concatenated LSU, ITS, and *TEF1*-*α* regions) and distinct morphological features. Giraldo & Crous [22] did not report mycelial ropes and coils in their isolates of *M. stromaticum*. However, we found that mycelial ropes and coils were common in all the isolates of *M. shandongensis* and *M. zingiberis*. Furthermore, both species display wider conidiophores, conidiogenous cells, and conidia compared to those of *M. stromaticum*.

*P. cucumerina*, the type species of *Plectosphaerella*, was previously identified as *Venturia cucumerina* from *Cucumis sativus* in Sweden [65]. The genus *Plectosphaerella* was later established by Klebahn [27] and was also obtained from *Cucumis sativus. Plectosphaerella* was placed in the family *Plectosphaerellaceae* based on phylogenetic analysis of the LSU locus [17]. Only three species of sexual morphs have been reported in *Plectosphaerellaceae* [22,66]. However, the asexual morphs are relatively homogeneous [16], and the main distinguishing characteristics of this genus depend on conidial shape and dimension as well as the occurrence or absence of chlamydospores [17,36]. Species of this genus have diverse nutritional modes and habitat sources, including plant pathogens, plant endophytes, animal pathogens and soil-borne saprobes [17,20,22,30,35,37,67,68]. This morphological simplicity and host variability hinder accurate identification, rendering traditional method insufficient [16,17,30]. While ITS and LSU loci are routinely employed for species delimitation of *Plectosphaerella* [17,20,69], their resolution is often inadequate due to the limited sequence divergence in this genus [17]. To improve taxonomic clarity, protein-coding genes such as *TEF*-*1α*, *CaM*, *TUB2*, and *RPB2* have been increasingly utilized in recent studies [22,32,37,66,70]. The combination *P. cucumerina*, introduced by Gams [38], is recognized as a cosmopolitan root-infecting pathogen with a broad host range, including *Apiaceae*, *Asteraceae*, *Brassicaceae*, *Cucurbitaceae*, *Fabaceae*, *Poaceae*, and *Solanaceae*. Compared with these previous studies, we isolated *P. cucumerina* from decaying rhizomes and roots of ginger. To our knowledge, this is the first documented isolation of *P. cucumerina* from ginger (*Zingiberaceae*) worldwide.

The pathogenicity trials revealed that all tested *Plectosphaerellaceae* spp. were pathogenic, though their virulence levels varied. Detached rhizomes inoculation provided initial evidence of pathogenic potential, which was further supported by symptoms development in intact seedlings inoculation. *G. serrae* exhibited the highest aggressiveness, triggering severe symptoms including rhizome browning, leaf yellowing, and subsequent plant death. These findings align with Giraldo and Crous [22], who reported similar symptoms on okra, amaranth, beet and potato. *M. shandongensis* and *M. zingiberis* caused rhizome browning and leaf curling of the ginger seedlings. Carlucci et al. [17], Raimondo [35] and Li et al. [40] associated symptoms with *P. cucumerina* such as stunting, root rot, leaf chlorosis, and stem base browning, with this seen as a necrotrophic pathogen in the present investigation. Recent research has demonstrated that *Gibellulopsis* sp. and *P. cucumerina* were enriched in diseased ginger crops, serving as potential biomarkers of rhizome rot disease [11]. Ginger diseases are generally categorized into two groups: aerial and soil-borne. Aerial diseases primarily include rust, anthracnose, and leaf spot [6,7]. The typical symptoms of these diseases appear as oval to irregular, chlorotic lesions on the leaves. These distinct lesions are clearly differentiated from the uniform chlorosis of entire leaves caused by soil-borne diseases. The major pathogens responsible for soil-borne diseases in ginger include *Fusarium* spp., *Pythium* spp., and *Ralstonia solanacearum* [5,8]. The four tested *Plectosphaerellaceae* spp. exhibit symptoms including rhizome and root rot, leaf yellowing, wilting, and subsequent plant death, which is closely similar to disease caused by *Fusarium* spp. However, *Plectosphaerellaceae* spp. infection does not induce severe wilting until the rhizome is completely rotten. In contrast, *Fusarium* spp. infection results in the entire tiller to die, accompanied by rapid wilting and desiccation of the leaves. Additionally, the symptoms caused by *Pythium* spp. appear on the above-ground part of the rhizome-stem interface as brown lesions that lead to stem rot, leaf chlorosis, and rhizome decay. Moreover, bacteria like *Ralstonia solanacearum* infection releases a pungent odor while *Plectosphaerellaceae* spp. infection does not result in any foul odor.

## 5. Conclusions

This study discovered and described two new species, one newly recorded species, and one new host record, causing rhizome and root rots of ginger in Shandong Province, China. These new findings significantly enrich our understanding of the biodiversity of the *Plectosphaerellaceae* family and provide valuable insights into the impact of diseases caused by these newly discovered pathogens on ginger production. Cultivation systems, such as continuous cropping in Shandong Province, may promote the development of ginger rhizome rot diseases. The infection processes and pathogenic mechanisms of the *Plectosphaerellaceae* species in causing ginger diseases remain largely unknown. Accurate identification of the causal agents and understanding their diversity through morphological, molecular, and pathogenic analyses are essential for effective disease management and the formulation of control strategies. Ongoing research into the comprehensive characterization of the *Plectosphaerellaceae* species causing this disease, including key virulence determinants and pathogenic mechanisms, will be crucial for developing targeted control measures and safeguarding ginger production in China and worldwide.

## Figures and Tables

**Table 2:** BLAST results for our strains SAAS 311704, SAAS 381402, SAAS 381414 and SAAS 481921.

Strain	Molecular Marker	Closest Species	Identity (%)	GenBank Accession Number	Identities
SAAS 311704	LSU	*Gibellulopsis nigrescens* CBS 179.40	100.00%	MH867573.1	851/851 (no gaps)
	ITS	*Gibellulopsis nigrescens* 74_ITS4	100.00%	OP498056.1	556/556 (no gaps)
	*TEF1*-*α*	*Gibellulopsis serrae* MFLUCC:23-0308	99.75%	PP866301.1	802/804 (no gaps)
SAAS 381402	LSU	*Musidium stromaticum* S20-1	100.00%	LC743850.1	818/818 (no gaps)
	ITS	*Musidium stromaticum* CBS 863.73	98.77%	MH860814.1	561/568 (2 gaps)
	*TEF1*-*α*	*Acremonium stromaticum* CBS 863.73	98.90%	LN810533.1	899/909 (no gaps)
SAAS 381414	LSU	*Musidium stromaticum* S20-1	99.50%	LC743850.1	795/799 (no gaps)
	ITS	*Musidium stromaticum* CBS 863.73	98.03%	MH860814.1	546/557 (no gaps)
	*TEF1*-*α*	*Acremonium stromaticum* CBS 863.73	97.84%	LN810533.1	904/924 (no gaps)
SAAS 481921	LSU	*Plectosphaerella cucumerina* CAES PC01	100.00%	MK143394.1	862/862 (no gaps)
	ITS	*Plectosphaerella cucumerina* FL08-0027	100.00%	AB469880.1	555/555 (no gaps)
	*TEF1*-*α*	*Plectosphaerella cucumerina* SKH23026	100.00%	PV593119.1	923/923 (no gaps)

## Data Availability

The original sequence data presented in the study are openly available in [NCBI] at [https://www.ncbi.nlm.nih.gov/].
